# Sabouraud dextrose agar for the diagnosis of fungal keratitis

**DOI:** 10.4103/0301-4738.71684

**Published:** 2010

**Authors:** Viroj Wiwanitkit

**Affiliations:** Wiwanitkit House, Bangkhae, Bangkok – 10160, Thailand

Dear Editor,

I read the recent publication on diagnosis of fungal keratitis with great interest.[1] Das *et al*. concluded that “This study recommends use of blood agar or chocolate agar for diagnosis of both bacterial and fungal keratitis in situations with limited resources.”[1] Omitting sabouraud dextrose agar (SDA) seems to be a challenge. In microbiology, SDA is used as a standard for fungus study.[2] The use of few samples in this study should be carefully considered. In addition, some fungi, especially for *Histoplasma* spp. cannot be successfully cultured via blood or chocolate agars.[3–4]

## References

[1] Das S, Sharma S, Kar S, Sahu SK, Samal B, Mallick A (2010). Is inclusion of Sabouraud dextrose agar essential for the laboratory diagnosis of fungal keratitis?. Indian J Ophthalmol.

[2] Odds FC (1991). Sabouraud(’s) agar. J Med Vet Mycol.

[3] Procop GW, Cockerill FR, Vetter EA, Harmsen WS, Hughes JG, Roberts GD (2000). Performance of five agar media for recovery of fungi from isolator blood cultures. J Clin Microbiol.

[4] Roberts GD, Washington JA (1975). Detection of fungi in blood cultures. J Clin Microbiol.

